# Technology and Inpatient Care: Addressing Communication Barriers and Social Isolation

**DOI:** 10.1177/23743735261458025

**Published:** 2026-06-02

**Authors:** Sama Amirkhani-Ardeh, Aderonke Agboji, Trina Fyfe, Tammy Klassen-Ross, Shannon Freeman

**Affiliations:** 1 School of Health Sciences, 6727 University of Northern British Columbia, Prince George, BC, Canada; 2 School of Nursing, 6727 University of Northern British Columbia, Prince George, BC, Canada; 3 Northern Medical Program, 6727 University of Northern British Columbia, Prince George, BC, Canada

**Keywords:** social isolation, older adults, communication, communication barriers, inpatients, hospital settings, technology, patient experience, alternative level of care (ALC), acute care settings

## Abstract

Social isolation and communication barriers are critical challenges faced by hospitalized patients, particularly those in long-stay, Alternate Level of Care (ALC), and acute care units. This scoping review of published literature aimed to answer the research question: How technologies including communication technologies are being used to address social isolation and communication barriers among hospitalized older adults, and what is known about their influence on patient experience. CINAHL, APA PsycINFO, PubMed, ScienceDirect, and Web of Science were searched for studies published from 2015–2025. Thirty-five articles met the inclusion criteria, including qualitative, quantitative, and mixed methods research. Key contributors to social isolation included restricted mobility, cognitive decline, and limited visitation and emotional wellbeing. Communication barriers stemmed from institutional routines, physical impairments, and lack of access to digital tools. These challenges are linked to negative outcomes such as depression, anxiety, cognitive deterioration, and diminished quality of life. Technology-based solutions, ranging from video calls and tablets to wearable devices demonstrate potential in bridging communication gaps and reducing social isolation. Video and phone call tools can improve communication and connection, but they do not work the same for everyone. Involving patients, caregivers, and healthcare staff in designing technology may help make these tools more useful, easier to use, and more sustainable in healthcare settings. This scoping review identifies the need for accessible and well-supported digital interventions to improve communication and well-being among older adult inpatients. Future research needs stronger study designs, bigger sample sizes, and interventions that are better matched to patients’ needs.

## 1. Introduction

Social isolation has become an increasing concern, particularly as modern lifestyles and healthcare environments limit opportunities for in-person interaction. This issue is especially pronounced among patients during hospitalization, who may face barriers such as mobility restrictions, and the loss of social networks. Prolonged social isolation has been linked to negative emotional and psychological outcomes, including loneliness, anxiety, and depression,^1-3^ While various factors contribute to this growing issue, the ways in which people engage with social connections are evolving. Hospitals, traditionally focused on physical health, must now recognize the vital role of social connectedness in patient recovery and well-being.^4,5^ Facilitating regular communication between patients and their loved ones; whether through in-person visits, phone calls, or digital technologies, can help mitigate the harmful effects of isolation and enhance emotional support. Integrating communication-friendly hospital policies and tools can bridge the gap between medical care and emotional well-being, ensuring that hospitalization does not become an isolating experience but rather one that fosters connection, comfort, and healing.^4,6^ Although communication technologies are increasingly present in healthcare, their specific role in addressing communication barriers and social isolation among older adults inpatients remains unclear. To strengthen the focus of this scoping review, the research question is stated: How technologies including communication technologies are being used to address social isolation and communication barriers among hospitalized older adults, and what is known about their influence on patient experience.

In Canada, ALC is the standardized designation given to patients occupying a bed in a facility when they no longer require the intensity of resources or services provided in that care setting and the majority are older adults (typically aged 65 years and older).^7,8^ According to a 2010 Canadian Institute of Health Information (CIHI) report, more than 92,000 hospitalizations and 2.4 million hospital days in Canada were classified as long-stay units in 2008-2009, representing 5% of all hospitalizations and 13% of all hospital days.^9^ Long-stay patients occupy 14% of hospital days and ∼5,200 acute beds daily in Canada, with a median stay of 26 days, reducing capacity and worsening emergency department crowding, surgical delays, and patient flow.^9^

In BC in 2013-2014, 4.5% of hospitalizations and 12.6% of hospital days were classified as long-stay units.^10^ BC had the third lowest percentage of long-stay units’ days among Canadian provinces in 2012. There is also regional variation in long-stay units’ prevalence within BC, with the percentage of long-stay units’ days ranging from 5% to 25% across the province. Between 2001 and 2014, rates of long-stay units’ cases and days remained relatively constant in BC, despite an aging population.^7,10^

Long-stay units increase patient risk of cognitive decline, functional loss, falls, infections, ulcers, and medication errors.^11-13^ For frail older adults, long stays heighten risks of rapid health decline, added morbidity, disability, acute care use, or early LTC admission.^10,14^ Prolonged long-stays often cause social isolation, worsening mental health, limiting communication, and reducing recovery and quality of life.^10^ Qualitative studies involving long-stay units’ patients, and their carers show that their experience during ALC stays were mostly negative.^15^ In one Canadian study of the experience of carers of long-stay units’ patients, carers noted that the quality of care declined after long-stay units’ designation and expressed frustration when the needs of the patient were neglected.^15^ Patients reported limited autonomy, poor communication, and isolation in hospital, often preferring residential care for greater engagement and reduced loneliness.^16^ Hospitals should enhance communication and support to strengthen older patients’ social connectedness and health outcomes.^16^ As more patients remain in hospitals for long periods, good communication between patients, families, and healthcare providers becomes increasingly important. Meaningful interactions help reduce stress, improve emotional well-being, and support patient autonomy. Using strong communication strategies whether in person, through programs, or with digital tools can reduce isolation and support patient-centered care. New technologies such as electronic health records, data systems, and telemedicine also help manage patients’ complex needs more effectively.^17^

Despite the growing number of patients in long-stay units in hospitals, there remains limited evidence on how these patients maintain communication and social connection during prolonged stays. To address this gap, this scoping review provides a comprehensive overview of existing evidence on communication practices and social isolation among hospitalized patients, focusing on their lived experiences and the role of technology in this context. It explores the barriers and facilitators that influence patients’ ability especially older adults to communicate and stay connected during hospitalization and identifies gaps in the literature to inform strategies that enhance communication, emotional well-being, and social connectedness in hospital settings.

## 2. Methodology

This scoping review followed a comprehensive methodology to map out the existing literature on how communication technologies are being used to address social isolation and communication barriers among hospitalized older adults. Arksey and O’Malley’s five-stage framework guided the review process: first, clearly defining the objective of the review to determine what the review aims to achieve. Second, identifying and selecting relevant literature involves choosing appropriate databases, keywords, and criteria to gather a comprehensive range of studies. Third and fourth, data extraction and charting the extracted data into categories or themes.^18^ Finally, collating, summarizing, and reporting the data to provide an overview of findings and trends.

The databases searched included CINAHL (EBSCO), APA PsycINFO (EBSCO), PUBMED, ScienceDirect, and Web of Science, chosen for their coverage of health sciences, psychology, and gerontology literature. The search was conducted using a combination of keywords and controlled vocabulary terms related to “Patient Experience,” “Older Adults,” “Communication,” “Hospital Settings,” and “Well-Being.” Keywords included terms like “Social Isolation,” “Patients Experience,” “Elderly,” “Long-Term Stay”, “Inpatients”., “Hospitalization”, “Loneliness”, “Geriatric,” “Mental Health,” “Physical Health,” and “Social Well-being.” Boolean operators (AND, OR) were used to combine keywords, ensuring a comprehensive search. In addition, this study is a scoping review of published literature. Ethical approval was not required.(Table 1)

**Table 1.** Keywords and Search Strategy in Databases

Category	Keywords and terms
Population	Older Adults, Elderly, Geriatric, Over 65
Intervention	Technology-Based Interventions, Digital Health, Telehealth, E-health, Mobile Health
Setting	Hospital Settings, Long-Term Stay, Alternative levels of Care (ALC)
Databases	CINAHL (EBSCO), APA PsycINFO (EBSCO), ScienceDirect, and Web of Science, PUBMED
Study Types	Original Research Articles (Quantitative Research, Qualitative Research, Mixed Methodes)
Outcomes	Well-Being, Social Isolation, Loneliness, Mental Health, Physical Health, Social Well-Being, Communication
Search Strategy	Boolean Operators (AND, OR)

**Figure 1.**
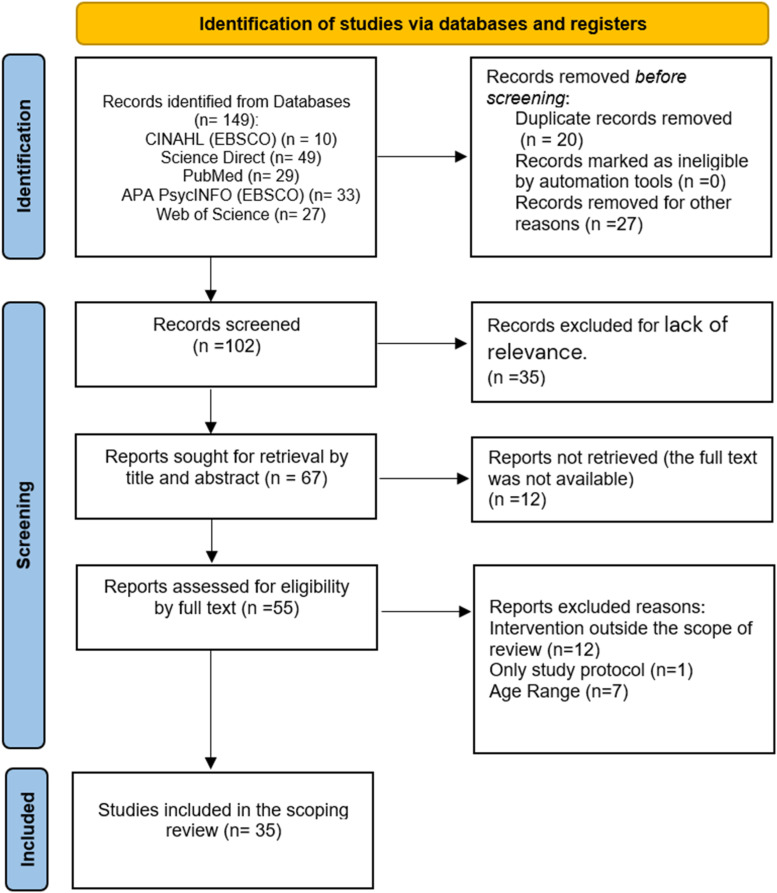
PRISMA-ScR flow diagram for the scoping review process

The scoping review included English-language articles published between 2015 and 2025 to ensure the inclusion of literature from the past ten years to capture recent developments in healthcare communication. Duplicates were removed using Zotero, and article selection was conducted in two stages: title/abstract screening and full-text review. Studies were excluded if they did not focus on patients experience, were outside care settings, lacked a technology intervention focus, were not in English, or were non-peer-reviewed materials. Non-English studies were excluded due to the lack of translation resources, this could reduce the accuracy of the data and make it harder to compare studies, so using consistent methods is important. However, this English-only restriction may have caused to miss relevant studies published in other languages, particularly from regions where digital health and aging research are rapidly growing. A technology intervention component was required for all included studies, as the review specifically focused on technology’s role in addressing communication barriers and social isolation. Only technology-focused studies were included because the aim of this review was to examine how technological tools influence communication, social connection, and patient experience in inpatient settings, ensuring that the scope remained aligned with the research question. The review process followed the Preferred Reporting Items for Systematic Reviews and Meta-Analyses extension for Scoping Reviews (PRISMA-ScR) checklist to ensure comprehensive and transparent reporting. A PRISMA-ScR flowchart was used to document record identification, screening, and inclusion, helping to highlight knowledge gaps and guide future research.^18^ The data were analyzed using descriptive statistics and thematic analysis to summarize study characteristics and key themes related to technology’s impact on patients’ well-being.(Figure 1)

## 3. Results

Out of the 35 articles reviewed, 16 were quantitative studies (reporting effect sizes or statistical significance where available), 7 were qualitative studies (summarizing thematic findings), and 4 were mixed studies (See Appendix A). The focus of the studies varied, with 12 articles intentionally studying older adult patients with various degrees of cognitive impairment, 14 articles focused on mental health among older adult patients, and 3 articles investigating physical injuries in older adult patients. However, there was a high prevalence of participants recruited with cognitive impairments and mental health issues across all the studies.

Geographically the studies were diverse. Five were conducted in the USA,^11,19-22^ four studies in the United Kingdom,^23-26^ Two studies were conducted in Hong Kong,^27,28^ and one each in Italy,^29^ Spain,^30^ Germany,^19^ Canada,^31^ Bangladesh,^32^ Sweden,^33^ Libya,^34^ and Switzerland.^35^ Two studies had an international perspective, analyzing different countries including Japan, Brazil, China, Switzerland, and the USA.^36,37^

Building on these descriptive characteristics, the included studies revealed several recurring concepts and patterns in relation to communication barriers and social isolation among older adults’ patients. The themes were interpreted through the lens of patient experience. The following section presents the key themes such as cognitive function, emotional wellbeing, mental and physical health identified through thematic analysis.

### 3.1 Technology and Physical Health Outcomes

Fifteen studies reported on the effects of technology on physical health. Positive effects included improved physical functioning and mobility through activities like fitness apps and virtual exercises,^38^ while negative effects included sedentary lifestyle issues,^39^ and musculoskeletal problems.^39^ This contrast suggests that the impact of technology on physical health may depend largely on the type and purpose of the intervention (such as communication and group activity, game, fitness), as well as the level of physical engagement encouraged. This supports literature indicating that while digital engagement can motivate physical activity through structured programs, prolonged screen use or lack of movement remains a concern, especially in hospital settings where mobility is already limited.^26^ These findings relate to patient experience through changes in mobility, comfort, and overall physical functioning. Interactive or movement-encouraging technologies supported better engagement in daily activities, whereas prolonged passive use contributed to decreased mobility and discomfort (See Appendix A).

### 3.2 Technology and Cognitive Experience in Older Adult Inpatient Care

Most studies focus on cognitive and mental health, highlighting these patients’ vulnerability to isolation and the value of communication for stability and engagement.^40^ Despite the importance of cognitive function in older adults, only 12 studies explored the relationship between using tablets and technology and cognitive function, reporting positive effects such as enhanced processing speed, improved memory and learning, and reduced cognitive decline (See Appendix A). The limited number of studies examining cognitive outcomes suggests that this area remains underexplored within the broader literature. These cognitive-related findings directly influence patient experience by shaping a patient’s ability to stay oriented, engaged, and emotionally regulated during hospitalization. Technologies that promote cognitive stimulation were consistently associated with improved engagement and reduced confusion among patients. Negative effects included attention deficit, impaired sleep, and potential digital addiction.^27,41^ This variation in outcomes suggests that the cognitive impact of technology may depend on factors such as patient cognitive status, duration of use, and type of digital engagement. These findings align with prior research suggesting that technology-facilitated communication and interactive digital tools can help maintain or enhance cognitive engagement in older adults when designed appropriately.^41^ Cognitive-related findings indicate that when designed appropriately, technology can contribute to a more stable and engaging patient experience.

### 3.3 Technology and Emotional Well-Being in Older Adult Inpatient Care

Despite the growing concern regarding behavioral and psychological symptoms (BPSDs) in patients over 65, only 13 out of the 35 articles addressed these issues in their outcomes (See Appendix A). While the effects of using tablets and technology on these symptoms were complex, several studies reported a significant relationship between technology use and increased willingness among patients. Factors affecting willingness included familiarity with technology, fear of technology, and cost.^23,27^ These findings suggest that patient readiness and acceptance may play a critical role in determining the effectiveness of technology-based interventions.

These findings suggesting that technology acceptance is strongly influenced by user comfort, perceived usefulness, and previous exposure to similar tools.^26^ Although the psychological well-being of patients is important for their quality of life, only 19 out of 35 studies investigated this aspect, with most reporting improvements in psychological QoL, including independence in daily activities.^40^ This reinforces prior studies indicating that digital communication tools can reduce depressive symptoms and loneliness in older adults, especially when they are used to maintain family or peer connections during hospitalization.^11^ In addition, within the studies investigating psychological well-being, subthemes included effects on depression, anxiety, loneliness, happiness, and personal development.^41^ The diversity of these outcomes highlights the multifaceted nature of emotional well-being but also complicates direct comparison across studies. The presence of these subthemes aligns with communication-focused literature that positions emotional and relational support as key outcomes of older adult patients’ access to virtual and interactive technologies.^10^

### 3.4 Technology and Social Connectedness

Technology via video calls, social media, gaming, and apps, reduced isolation and improved cognition, activity, and healthcare access.^28,34^ These findings are consistent with previous studies that have highlighted the role of communication technologies in fostering social connectedness and reducing loneliness among hospitalized or long-term care patients, particularly older adults.^40^

The literature review suggests a diverse range of studies exploring the impact of technology on various aspects of patients’ well-being, with a general trend toward positive outcomes. However, it also emphasizes the need for a balanced and mindful approach to technology use, considering individual differences and potential risks associated with excessive usage.^11^ The findings show that virtual social interaction helps older adult patients feel less isolated, stay connected with their families, and feel more emotionally stable. These benefits appear particularly relevant in hospital settings where in-person visits may be limited. Communication technologies may function as social bridges, especially for older adults in hospitals who cannot have many visitors, although their effectiveness may depend on accessibility, patient comfort with technology, and level of support provided.

### 3.5 Technology and Patient Experience Outcomes From a Co-Design Perspective

Diverse technological applications included the use of wearable facilities, smartwatches, smart glasses, games, touchscreen monitors, mobile phones, gadgets, laptops, and tablets.^27,34^ The sample sizes of the studies ranged from 14 to 1349 participants, with the majority having less than 100 participants.^38,42^ Tablets and other devices were used to enhance the connectedness and mental health of older adult patients.^38,42^ Synthesizing across the studies, communication technologies consistently influenced patient experience outcomes particularly emotional support, reduced agitation, improved connection with family/caregivers, and enhanced perceived quality of life. Devices enabling real-time communication (e.g., video calls) were frequently linked with reduced loneliness, better mood, and strengthened relational bonds during hospitalization (See Appendix A). However, the strength and consistency of these outcomes varied depending on technology intervention type, duration of technology use, and level of support provided by hospital staff or other caregivers. A range of measurement tools were employed to assess participant outcomes, including the Montreal Cognitive Assessment (MoCA), Cohen-Mansfield Agitation Inventory (CMAI), Geriatric Depression Scale (GDS), Activities of Daily Living (ADL), Quality of Life in Alzheimer’s Disease (QOL-AD), Medication Adherence Self-Efficacy Scale (MASES), Short Form 36 Health Survey (SF-36), and the Technology Acceptance Model (TAM).^24,27^ Regarding the type of studies, only one of the provided articles used an experimental design,^43^ which required the random allocation of participants to control and experimental groups. Instead, quasi-experimental designs are present in 11 studies (1, 3, 4, 6, 10, 12, 13, 16, 17, 19, and 22) (See Appendix A). Although these studies involve interventions or treatments, they do not include the random assignment of participants that characterizes true experimental research. On the other hand, non-experimental design is employed in 10 studies (2, 5, 8, 9, 11, 14, 15, 18, 20, and 21) (See Appendix A). These studies focus on observing and describing variables without manipulating them, often utilizing methods such as surveys, questionnaires, or analyzing existing data (See Appendix A). The dominance of non-randomized studies limits causal conclusions but still provides valuable insights into how patients perceive and experience technology in daily care. Also, across the included studies, interest holder engagement particularly the involvement of patients, caregivers, and frontline staff, played a key role in shaping the relevance, usability, and acceptance of technology-based interventions.^13^ This co-design approach may enhance technology- based intervention suitability and patient experience; however, its implementation and reporting varied across studies, making it difficult to determine its impact on outcomes.

## 4. Discussion

The discussion addresses three themes: intervention diversity and mixed evidence, engagement and co-design, and methodological gaps that shape the findings, influences, and future implications. Each of these themes will be elaborated on in the following paragraphs.

### 4.1 Technology’s Impact on Older Adults’ Experience

The geographical distribution of the included studies was broad, encompassing regions such as Hong Kong, Bangladesh, in Asia, Libya in North Africa, the United Kingdom, Italy, Spain, Germany, Sweden, and Switzerland in Europe, Canada in North America, and the United States of America. This global representation underscores the widespread interest in leveraging communication technologies to improve the well-being of older adult patients in various healthcare settings.

The utilization of technology in the reviewed studies was diverse, encompassing a wide range of devices and platforms, including wearable devices, smartwatches, smart glasses, games, touchscreen monitors, mobile phones, laptops, and tablets. Sample sizes varied significantly, ranging from 14 to 1349 participants. However, this diversity also brings challenges. The wide variation in intervention types of technologies, outcome measures, and patient populations (many studies under 100 participants) makes it difficult to compare studies directly and limits the ability to draw strong conclusions about effectiveness across healthcare environments.

Some studies suggest that using communication technologies such as video calling platforms or hospital-based chat applications might slow down mental decline and help people feel more connected, which could reduce feelings of loneliness and low self-esteem.^44^ Another assessment focuses on the effects on patients’ cognitive decline, loneliness, and self-esteem. Despite expectations, their results indicate no significant effects of the tablet intervention on these variables. The mixed results may be because the studies were not conducted under the same conditions, some interventions lasted longer, some older adult patients had different cognitive abilities, and some were more comfortable with technology than others. The results were also unclear because many studies had small sample sizes, making it hard to find real effects. These findings caution policymakers against being overly optimistic about communication technologies and enhance our understanding of mechanisms that may impede positive outcomes from ICT programs for patients.^41^

### 4.2 Interest Holder Engagement and Co-Design

Engaging patients, caregivers, and care providers in co-designing strategies to address long-stay units’ challenges is critical to ensuring care gaps are addressed and services align with needs. Co-design involves collaborating with end users and providers to design services, programs, or interventions aiming to enhance care quality. Emerging evidence suggests that involving patients and caregivers as partners can lead to improved care and outcomes.^13^ For example, Kuluski et al. co-designed an Alternate Level of Care (ALC) intervention by working directly with patients, caregivers, and providers to identify barriers to discharge and develop patient-informed solutions. The co-designed intervention included a communication guide to support ongoing conversations between patients, caregivers, and care providers, as well as a set of recommended services such as daily mobilization, tailored psychosocial activities, and a bedside storyboard to improve engagement.^13^ Despite this, only a small number of studies in this review incorporated meaningful co-design processes, which limits understanding of how patient-led or caregiver-led input may influence intervention success (See Appendix A). Future research should prioritize co-design to strengthen relevance and acceptability.

### 4.3 Limitations and Future Research Directions

In research involving older adults in healthcare environments, several studies that explored the use of communication technologies to support older adults’ well-being had small sample sizes or did not include well-controlled trial designs. This may suggest some limitations in the strength of the available evidence and may reduce confidence in the reported effectiveness of these interventions. These challenges may be related to differences in ethical requirements, data protection rules, and institutional approval processes across countries, and financial constraints, which can make it more difficult to implement controlled technology-based studies in healthcare settings. Many existing studies rely on quasi-experimental or observational designs, which limit causal interpretation and increase the risk of bias, as these approaches are often more feasible and easier to implement in real-world healthcare settings than fully randomized controlled trials.

Several studies reported positive effects of communication technologies on reducing depressive symptoms among older adult patients; however, the relationship between technology use and depression may appear to be influenced by other factors such as cognitive level of patients (See Appendix A). The cognitive level may refer to an individual’s cognitive abilities, including memory, attention, and problem-solving skills.^45^ Research suggests that patients with different cognitive profiles may respond differently to communication platforms. This may be because individuals with intact cognitive functioning may benefit more from certain types of communication platforms compared to those with cognitive impairments such as dementia. The lack of standardized outcome measures and consistent intervention protocols made it difficult to determine whether differences in results were due to the technology itself or contextual and methodological factors.

In this study, the inclusion criteria focused on peer-reviewed, English-language studies involving older adults in healthcare environments, and communication technologies interventions. While this approach ensured relevance and feasibility, it may have resulted in the exclusion of relevant grey literature or studies conducted in non-English contexts that could have provided additional insights and potentially strengthened the overall evidence base. Future research could benefit from more rigorous study designs, larger sample sizes, and clearer technology-specific intervention protocols (e.g., frequency and duration of technology use, type of communication platform, level of staff or family support, and training provided to older adults) to help strengthen the current evidence base.

## 5. Conclusion

Long-stay hospitalization increases communication challenges due to sensory decline, cognitive impairments, and the hospital environment, which can lead to frustration and social isolation especially for older adult patients. This review highlights that communication technologies are being introduced as potential strategies to support connection and emotional well-being among hospitalized older adults. Technology-based interventions show promise; however, the current evidence is limited and should be interpreted carefully due to small sample sizes, which may weaken the statistical power of the studies, the limited number of randomized study designs, which may reduce the strength of the evidence, and differences in the types of interventions used (such as regular calls, video calls, and social apps), as the lack of a consistent methodological protocol across studies made comparison of the evidence somewhat challenging. Outcomes may also vary depending on patients’ cognitive status, how well the technology matches their needs, and the level of support from staff and family members. Mobility, cognitive symptoms, or overall well-being of older adult patients may improve; however, these effects are not consistent across all individuals. Tools such as video and telephone calls may enhance communication and social connection, although outcomes may vary depending on patient characteristics and healthcare environments. Future research should use stronger study designs, such as more randomized studies, including larger samples, and developing interventions that are tailored to the specific needs of older adult inpatients. This review synthesizes existing evidence on how communication technologies are being applied within older adults’ healthcare environments. As this is a review of existing literature, these conclusions represent synthesized findings rather than new empirical data. Further high-quality research and continued evidence synthesis are needed to support reliable and practical improvements in healthcare practice.

## Supplemental Material

Supplemental Material - Technology and Inpatient Care: Addressing Communication Barriers and Social Isolation Supplemental Material for Technology and Inpatient Care: Addressing Communication Barriers and Social Isolation by Sama Amirkhani-Ardeh, Aderonke Agboji, Trina Fyfe, Tammy Klassen-Ross, Shannon Freeman in Journal of Patient Experience
