# Supplementary material for: Technology and Inpatient Care: Addressing Communication Barriers and Social Isolation
Source: J Patient Exp. 2026 Jun 2;13:23743735261458025. doi: 10.1177/23743735261458025 (PMC13234491; doi:10.1177/23743735261458025)
Supplement: Supplemental Material - Technology and Inpatient Care: Addressing Communication Barriers and Social Isolation [file sj-pdf-1-jpx-10.1177_23743735261458025.pdf]

Supplementary File – Appendix A

Title	Methods	Research Location	Purpose	Tech used	Number of participants	Measurement tools	Results
Tong T, et al. A serious game for clinical assessment of cognitive status: validation study. JMIR Serious Games. 2016;4(1):e7. doi:10.2196/games.5006	quantitative/a prospective observational clinical study	Toronto, ON, Canada	The study aims to test the feasibility of using a tablet-based game for cognitive assessment in older adults and to preliminarily validate it against standard mental status tools. P1	using computers and tablets, use of a serious game	older adults (N=146; age: mean 80.59, SD 6.00, range 70-94 years)	Mini-Mental State Examination (MMSE), Montreal Cognitive Assessment (MoCA), and the Confusion Assessment Method (CAM)	This research demonstrates the feasibility of using serious games in a clinical setting. Further research is required to demonstrate the validity and reliability of game-based assessments for clinical decision making.
Omar KS, et al. An Intelligent Assistive Tool for Alzheimer's Patient. In: 2019 1st International Conference on Advances in Science, Engineering and Robotics Technology (ICASERT). IEEE; 2019:1-6. doi:10.1109/ICASERT.2019. 8934542	quantitative	Bangladesh	The objective of this research is to propose an assistive tool for Alzheimer's patients and their caregivers to provide support like health monitoring, assist to find lost items, provide reminder to take medicine and assist to monitor patient's location. P8	An assistive tool was developed with hardware components and a mobile app built on the Ubidots cloud platform.	A light-weighted evaluation study was carried out with 15 participants	an intelligent assistive tool for Alzheimer's patients as shown in Fig. 1. The framework consists of four modules: heart rate monitoring, lost item finder, smart medicine box and lost patient locator.	The evaluation study showed that the proposed system was effective and usable for the patients<br>and their caregivers.
Chen K, et al. A Tablet-Based Volunteer-Mediated Intervention for Cognitively Impaired Older People: A Pretest–Posttest. Res Soc Work Pract. 2020;30(3):288-297. doi:10.1177/1049731519863 103	qualtitative	Hong Kong	This study evaluated the potential effectiveness of a tablet-based, volunteer-led intervention (Lok Chi In-home Training) for cognitively impaired older people in improving cognitive and emotional health. P259	tablet	involving 57 community-dwelling older people with cognitive impairments (Montreal Cognitive Score between 13 and 22, without neuropsychiatric behavioral problem) aged over 60	Montreal Cognitive Score between 13 and 22, without neuropsychiatric behavioral problem	This study demonstrated the feasibility and potential benefits of Lok Chi intervention for improving cognition and emotion.

Chiu CJ, et al. The attitudes, impact, and learning needs of older adults using apps on touchscreen mobile devices: results from a pilot study. Comput Human Behav. 2016;63:189-197. doi:10.1016/j.chb.2016.05.020	Mixed-method approach	Taiwan	This study describes an embedded mixed-method research evaluation of an eight-week touchscreen mobile device training for thirty-nine older adults who were recruited from a community center in a low Internet usage area in southern Taiwan. P189	touchscreen-based apps	The participants were recruited from a community via flyers. Thirty-nine older adults (25women,14men), aged 50 or over, signed up for the training course.	This study used a mixed method embedded design to examine whether training on Internet use and touchscreen applications could improve older adults' attitudes and psychological well-being. The research protocol received IRB approval before the study began.	the possibility of touchscreen-based apps, including health-, entertainment-, transportation-, and social media-related apps, for improving psychological well-being in older adults with limited Internet experience living in the community. Discussion on their learning needs was also provided.
Begde A, et al. Exploring factors influencing willingness of older adults to use assistive technologies: evidence from the cognitive function and ageing study II. Inf Commun Soc. Published online May 1, 2023:1-18. doi:10.1080/1369118X.2023.2205915	quantitative	Nottingham, UK	This analysis used data from the Cognitive Function and Ageing Study (CFAS-II), which examined brain health in older adults. The study included questions on access to and interest in internet technologies. Binary logistic regression was applied to identify factors influencing willingness to use technologies supporting memory and daily living (ADL). P10	laptops and tablets	541 people aged 75 years and older from Cambridgeshire, Nottingham and Newcastle responded.	used data collected in the Cognitive Function and Aging Study (CFAS)-II ; The participants were randomly selected from the general practitioner list and their physical, mental, and cognitive functions were assessed, as well as their socio-demographic characteristics, by a trained researcher using a detailed questionnaire.	Older people, females and those with less access to technologies should be considered as target groups by healthcare providers, policymakers, and technology producers to promote technology and support healthy and independent ageing.
Andrews JA, et al. Older adults' perspectives on using digital technology to maintain good mental health: interactive group study. J Med Internet Res. 2019;21(2):e11694. doi:10.2196/11694	qualitative	United Kingdom	This study sought to explore older adults' perspectives on technology to support good mental health. P21	apps and websites	A total of 15 older adults aged 50 years or older, in two groups, participated in sessions to explore the use of digital technologies to support mental health.	The COBALT tool kit uses techniques; qualitative research approach	Older adults are motivated to use digital technologies to improve their mental health, but barriers remain that developers need to address for this population to access them.

Kerkhof Y, et al. Randomized controlled feasibility study of FindMyApps: first evaluation of a tablet-based intervention to promote self-management and meaningful activities in people with mild dementia. Disabil Rehabil Assist Technol. 2022;17(1):85-99. doi:10.1080/17483107.2020.1765420	mixed methods	Netherlands	This study examined the feasibility, implementation, and impact of FindMyApps, a tablet-based intervention for people with dementia.<br>The tool helps users find suitable apps for self-management and meaningful activities. It also trains informal carers to use errorless learning techniques to support tablet and tool use. P85	tablet training	Twenty persons with mild dementia and carer dyads were randomly assigned to the FindMyApps group, (n=10) receiving either the FindMyApps training and selection tool, or a control condition, (n=10) receiving only a short tablet training.	The FindMyApps	Qualitative results indicate that the FindMyApps intervention has the potential to positively influence the self-management abilities and engagement in meaningful activities of people with dementia. Remarks are made to improve the intervention, and recommendations are given for future effectiveness studies.
Cid A, et al. Tablets for deeply disadvantaged older adults: Challenges in long-term care facilities. Int J Hum-Comput Stud. 2020;144:102504. doi:10.1016/j.ijhcs.2020.102504	Quantitative	Montevideo, Uruguay	Research suggests that technology might lead to a slowdown in cognitive deterioration, and could create opportunities for social connectedness, helping alleviate social loneliness and low self-esteem. P102	tablets	the 93 participants were 78 years old average.	tablet is a touchscreen (8″) mobile device that runs an Android operating system	Results suggest no significant effects of the tablets intervention on these variables. Our findings are important in the avoidance of techno-optimism among policy makers and the understanding of mechanisms that may prevent positive results from ICT programs for the elderly.
Ferm U, et al. Tablet computer-supported conversation between people with dementia and their carers: technology as interactional focus. Univ Access Inf Soc. 2021;20(4):753-765. doi:10.1007/s10209-020-00745-4	Quantitative	Sweden	to explore when and how technology becomes a topic in interactions involving people with dementia and their carers. P753	using tablet computers and two web-based applications	Three dyads of older women with dementia and their carers participated in the study. over 65 years old and lived in care homes	Two readymade and earlier tested applications called CIRCA and CIRCUS were used in the study	If people with dementia and their carers should benefit from today's technology, such as there is evidence for the interactions examined in this study, their homes and daily environments must be equipped with sufficient internet access and technical support.

Vaportzis E, et al. A Tablet for Healthy Ageing: The Effect of a Tablet Computer Training Intervention on Cognitive Abilities in Older Adults. Am J Geriatr Psychiatry. 2017;25(8):841-851. doi:10.1016/j.jagp.2016.11.015	quantitative	Edinburgh, UK	To test the efficacy of a tablet computer training intervention to improve cognitive abilities of older adults. P841	tablets	The study began with 48 participants, but 5 withdrew, leaving 43 older adults (ages 65–76) in the final analysis. Of these, 22 were assigned to the tablet intervention group and 21 to a no-contact control group. All participants were relatively healthy and community-dwelling.	A battery of cognitive tests from theWAIS-IV measuring the domains of Verbal Comprehension, Perceptual Processing, Working Memory, and Processing Speed, as well as health, psychological, and well-being measures	Tablet training, as a mentally challenging activity, was linked to improved processing speed in older adults. Learning new skills later in life, including technology use, may help reduce or delay age-related cognitive decline. Such skill development can also support everyday functioning and enhance quality of life.
Menghi R, et al. Product Service Platform to improve care systems for elderly living at home. Health Policy Technol. 2019;8(4):393-401. doi:10.1016/j.hlpt.2019.10.004	quantitative	Italy	Governments are promoting ageing-in-place to reduce healthcare costs and improve services. ICT solutions are being developed to support elderly home care, but much research remains technology focused. Crucially, many studies overlook the real needs and perspectives of older adults and other stakeholders in healthcare. P393	computers, tablets, mobiles	The study involved 1346 of the 2459 inhabitants over the age of 75 of eight municipalities of the inner areas of the Marche region (Italy), which are affected by depopulation and lack of territorial enhancement.	the PSP services/Several studies have emphasized the potential benefits of using ICT, particularly for the elderly	Including end-users and stakeholders enabled multiple perspectives and the creation of a value network that integrates existing and new resources. The study offers guidelines for developing platforms that leverage stakeholder strengths without adding barriers.
Ke C, et al. Changes in technology acceptance among older people with dementia: the role of social robot engagement. Int J Med Inf. 2020;141:104241. doi:10.1016/j.ijmedinf.2020.104241	quantitative	Hong Kong	Social robots can reduce loneliness and agitation in people with dementia, but acceptance remains low. This study investigated how direct interaction with the humanoid robot Kabochan affected technology acceptance among long-term care residents with dementia. P104	smartphones and tablets	A total of 103 residents clinically diagnosed with dementia, with a mean age of 87.2 years (SD = 7.4)	the randomised controlled trial (RCT)/Montreal Cognitive Assessment 5-minute Protocol (MoCA),	Exposure to Kabochan improved residents perceived ease of use of technology but not other attitudes or beliefs. Direct engagement with humanoid robots shows promise for enhancing technology acceptance in dementia care.

Lindquist LA, et al. Rationale and study design for decision making & implementation of aging-in-place/long-term care plans among older adults. Contemp Clin Trials Commun. 2021;22:100756. doi:10.1016/j.conctc.2021.100756	quantitative	Chicago, IL, USA	We are testing how decision-making and planning for AIP(aging-in-place) is impacted by changes in older adults' cognition and function, chronic conditions, social influences, environmental factors and identifying the mediating/moderating interactions between factors. We will also assess whether decision-making and planning for AIP translates into timely adoption of plans and goal concordance between older adults and their surrogate/caregiver decision makers. P100	online intervention/ computers and laptop	Initially a cross-sectional study (LitCog I), 900 adults ages 55–74 were recruited in 2008 from 8 community-based, primary care practices in Chicago.	The study protocol was approved by the Northwestern University Institutional Review Board, and is registered on Clinicaltrials.gov (NCT03960476)/Cognitive Function among Older Adults (LitCog) research study (R01AG03611) that involves extensive cognitive testing	This study examines how age-related cognitive, affective, social, and motivational changes influence decision-making around aging-in-place. It will assess how social, environmental, and personal factors moderate both decision-making and implementation. Using longitudinal data from the LitCog cohort, it will explore how changes in cognition, health, and social contexts shape aging-in-place decisions and interpersonal influences.
Chu CH, et al. The Impact of COVID-19 on Social Isolation in Long-term Care Homes: Perspectives of Policies and Strategies from Six Countries. J Aging Soc Policy. 2021;33(4-5):459-473. doi:10.1080/08959420.2021.1924346	qualitative	Belgium	an international perspective. For example, Brazil and China are developing their LTC sectors whereas the LTC sectors in Canada, Japan, Switzerland and the U.S are already well established. P459	Mobile/tablets/laptop	The narrative article,and not to explicitly mention the population	the WE-THRIVE initiative to measure person-centered care outcomes and may be appropriate to use in the future for cross-comparative work	International visitation restrictions in long-term care homes during COVID-19, though well-intentioned, worsened pre-existing social isolation. This perspective reviews LTC policies from six countries and their impacts. It also proposes five strategies to mitigate social isolation, relevant both during and beyond the pandemic.

Latifovic E, et al. Intercultural communication in long-term care: The perspective of relatives from Switzerland. Z Für Evidenz Fortbild Qual Im Gesundheitswesen. 2023;178:1-7. doi:10.1016/j.zefq.2023.02.002	qualitative	Zurich	the aim of the present study was to define recommendations by interviewing relatives which can be used to promote intercultural communication in long-term care with regard to relationship building. P1	the content-structuring method with an inductive approach	(n = 14) of residents from two retirement centers in Switzerland	The first author personally transcribed the interviews, incorporating memos and field notes for analysis. Using MAXQDA (2020), the data underwent open coding, with sentences categorized into initial subgroupings.	Intercultural communication means being aware of the significance of culture and one's own origins to recognize the peculiarities of the counterpart and to interact sensitively with them. Relatives want to be actively involved when nurses reach their limits in communication and interaction. Relatives want safe care that is based on empathy. When nurses show concern towards others in a person-centered way and are aware of where they come from, this can promote trust and contribute significantly to supporting communication and interaction between cultures.
Embarak F, et al. Design of autonomous online social community architecture for older adults. Comput Electr Eng. 2022;100:107900. doi:10.1016/j.compeleceng.2022.107900	quantitative	Libyan village, the University of Benghazi, and a hospital	Here, a unique model is proposed which showcase the aged people to manage their own life. The tasks of caretakers are often complex, so it is essential to develop a mechanism that can render the support for satisfaction in un met daily needs of aged people. It aims at the aged people to accomplish their unfulfilled daily needs. P10	Design a unique model/compuers and Database servers	The study conducted interviews with 20 participants, including 10 elders (65+), caregivers, physicians, and community leaders, lasting 10–27 minutes. Among them were 9 females and 6 males, as well as relatives, neighbors, and friends involved in elder care. A questionnaire was also distributed to 40 individuals (21 males, 19 females), mainly men aged 60–69.	The framework is based on the web-based architecture. It includes a responsive UI model, which can be worked on the two PCs and cell phones.	In this research work, the architecture, design, and care support system has been presented to assist the elderly people. The expert review analysis shows that this system can consistently assist both the elders as well as the supporting communities with the requirements of the users. It also fills the gap in the existing elderly care systems. In addition, it can be accessed from the mobile as well as the web. The functional components used in this model provide more functional to the support for the system development team, based on their potential interest.

Bozan K, et al. How can technology enhance elderly adherence to self-managed treatment plan? Procedia Comput Sci. 2018;141:472-477. doi:10.1016/j.procs.2018.10.140	quantitative	USA	This study investigates the effect of information systems on retaining motivation to adhere to self-management and satisfaction with treatment. P472	application was available on a tablet, phone, or computer and users were provided with a brief user manual.	Participants were older adults, 65 years or older, with adequate computer experience to open an application or webpage and click with a mouse. A total of 78 participants were recruited for the study, and 12 dropped out.	The self-management app was accessible via tablet, phone, or computer, with a brief manual provided for users. Participants submitted their data, which could be viewed digitally or printed. Behavioral intention to continue using the app was assessed using adapted UTAUT intention-to-use measures.	The study recruited 78 participants, with 12 dropping out, leaving 24 in the treatment group and 22 in the control group who completed both surveys. Using Welch's two-sample t-test, goal-setting scores were compared at baseline and after 6 weeks. Results showed no difference initially, but after 6 weeks the treatment group (with feedback) had significantly higher goal-setting scores than the control group.
Koskas P, et al. Effect of a multi-domain intervention on the quality of life in older adults with major neurocognitive disorder: a pilot study. Rev Neurol (Paris). 2022;178(4):355-362. doi:10.1016/j.neurol.2021.06.010	quantitative	France	A 3-month personalized multi-domain intervention at Bretonneau Hospital aims to improve quality of life for older adults with major neurocognitive disorders, where occupational therapy and cognitive stimulation alone have shown limited efficacy. P355	computers/laptops-rehabilitation day care unit (RDCU) program	60 outpatients (mean age 83.3 ± 5.8 years, with 70% women)	-Mini-Mental State Examination (MMS)<br>-Instrumental Activities of Daily Living (IADL), Activities of Daily Living (ADL)<br>-Neuropsychiatric Inventory (NPI)<br>-QoL-Alzheimer's Disease (QoL-AD) scale (pre–post comparison)	The study at Bretonneau Hospital's Day Care Unit (Paris, France) included 60 outpatients (mean age 83.3 years; 70% women). A significant improvement in QoL-AD scores was observed after the 3-month intervention (31.8 ± 4.9 → 32.9 ± 5.2; P = .008). Patients who benefitted most were older and had lower baseline QoL, with no other characteristics linked to improvement.
Tong T, et al. Test-Retest Reliability of a Serious Game for Delirium Screening in the Emergency Department. Front Aging Neurosci. 2016;8. doi:10.3389/fnagi.2016.00258	Quantitative	Toronto, Canada	To demonstrate the test-retest reliability of the screening tool over time in a clinical sample of older emergency patients. A secondary objective is to assess whether there are practice effects that might make game performance unstable over repeated presentations. P58	computer	A total of 114 patients participated in the study,between the ages of 70 and 104 years (SD=7). There were 61 females, and 53 males in the sample.	MMSE, and MoCA	A total of 114 adults (61 females, 53 males) between the ages of 70 and 104 years (M D 81 years, SD D 7) participated in our study after screening out delirious patients. We observed a test-retest reliability of the serious game (as assessed by correlation r-values) between 0.5 and 0.8 across adjacent sessions.

Ono T, et al. Association between meaningful activities at home and subjective well-being in older adults with long-term care needs: a cross-sectional study. Geriatr Nurs. 2023;52:121-126. doi:10.1016/j.gerinurse.2023.05.013	quantitative	Japan	We distributed a self-administered questionnaire to long-term care facilities in Japan and performed a linear mixed-effects model regression analysis of the responses. P121	TV, Music player, computers, laptop	the range of the target sample size to be 187-243. At one month before the start of the survey, the number of individuals who had registered from all study facilities was 425.	use of information and communication technology (ICT), self-rated health, instrumental self-maintenance intellectual activity, and self-rated-curiosity.	we found that both number of meaningful home activities (B=0.43; 95%CI :0.17, 0.70) and its interaction with preference(B=0.43;95%CI:0.79,0.08) we reassociated with SWB. These results suggest the importance of engaging in meaningful activities at home for older adults who do not work out. We should encourage older adults to participate in activities that match their preferences.
Kuo MH, et al. Using information and mobile technology improved elderly home care services. Health Policy Technol. 2016;5(2):131-142. doi:10.1016/j.hlpt.2016.01.005	quantitative	Taiwan	researchers have suggested applying information and mobile communication technology (IMCT) to enhance the utilization of elderly home care services. P131	mobile/tablets/laptop/computers	102 patients	The care service system was developed using AppServ (Apache, PHP, MySQL) and Microsoft Dynamics CRM for patient management.<br>A PHP plug-in with Google Maps API enabled care route planning.<br>Home care nurses accessed functions like route planning, health evaluation, and service logging via smartphones on Android, iOS, or Windows Mobile.	We conducted a user experience interview to document and analyze the feasibility and efficiency of the system. The results showed that users found the system very useful in improving the home care services. Particularly, the care scheduling, care management, tour planning, and service logging functions are the most useful features of IMCT tools for improving the home care services. However, the small mobile phone screen and function integrations were main constraints in the system usability.

Schuster AM, et al. COVID-19's Influence on Information and Communication Technologies in Long-Term Care: Results From a Web-Based Survey With Long-Term Care Administrators. JMIR Aging. 2022;5(1):e32442. doi:10.2196/32442	qualitative	United States	This study explored ICT access and use in LTC facilities and how LTC facilities adapted to try to enhance social connections for their residents during the COVID-19 pandemic. P22	information and communication technologies (ICTs)	70	a web-based survey	Since March 2020, a total of 53% (37/70) of the LTC facilities have purchased ICTs for residents' use. ICTs have mainly been used for videoconferencing with family members (31/36, 86%), friends (25/36, 69%), and health care providers (26/36, 72%). NHs were 10.23 times more likely to purchase ICTs for residents' use during the COVID-19 pandemic than ALFs (odds ratio 11.23, 95% CI 1.12-113.02; P=.04). Benefits of ICT use included residents feeling connected to their family members, friends, and other residents. Barriers to ICT use included staff not having time to assist residents with using the technology, nonfunctional technology, and residents who do not want to share technology.
Wilson R, et al. Care staff perspectives on using mobile technology to support communication in long-term care: mixed methods study. JMIR Nurs. 2020;3(1):e21881. doi:10.2196/21881	mixed methods approach (qualitative and quantitative)	Canada	This study aims to identify care staff's perspectives on the different ways of using devices and apps to support everyday communication with adults living in LTC homes and the priority care areas for using mobile technology to support communication with residents. P19	about ways of using mobile technology with residents	13	Concept mapping includes 2 main data collection phases: (1) statement generations through brainstorming and (2) statement structuring through sorting and rating.	Participants generated 67 unique statements during the brainstorming session. Following the sorting and rating of the statements, a concept map analysis was performed. In total, 5 clusters were identified: (1) connect, (2) care management, (3) facilitate, (4) caregiving, and (5) overcoming barriers. Although all 5 clusters were rated as useful, with a mean score of 4.1 to 4.5 (Likert: 1-5), the care staff rated cluster 2 (care management) as highest on usefulness, practicality, and probable use of mobile technology to support communication in LTC.

Currie M, et al. Attitudes towards the use and acceptance of eHealth technologies: a case study of older adults living with chronic pain and implications for rural healthcare. BMC Health Serv Res. 2015;15(1):162. doi:10.1186/s12913-015-0825-0	mixed-methods study	UK	A variety of eHealth initiatives (for example Pathways through Pain an online course aimed to aid self-help amongst those living with persistent pain) have been launched across the UK but roll out remains at an early stage. P13	email, mobile phone	168	Semi-structured interviews	People suffering from chronic pain access healthcare in a variety of ways. eHealth technology use was most common amongst older adults who lived alone. There was broad acceptance of eHealth being used in future care of people with chronic pain, but older adults wanted eHealth to be delivered alongside existing in-person visits from health and social care professionals.
Choi NG, et al. Telehealth use among older adults during COVID-19: associations with sociodemographic and health characteristics, technology device ownership, and technology learning. J Appl Gerontol. 2022;41(3):600-609. doi:10.1177/07334648211047347	qualitative	United States	In this study, they (1) examined rates and correlates of telehealth (video call) use among those aged 70+, and (2) tested the significance of access to information and communication technology (ICT) device ownership and knowledge of how to use the internet and devices as telehealth-enabling factors. P600	The Behavioral Model of Health Services Use served as the conceptual framework	139	interview	Results show that telehealth use increased to 21.1% from 4.6% pre-pandemic. In logistic regression models without technology-enabling factors, older age and lower income were negatively associated with telehealth use; however, when technology-enabling factors were included, they were significant while age and income were no longer significant. Ensuring that older adults have ICT devices and internet access may reduce health disparities and improve telehealth care delivery.
Wang S, et al. Technology to Support Aging in Place: Older Adults' Perspectives. Healthcare. 2019;7(2):60. doi:10.3390/healthcare7020060	qualitative	United States	The U.S. population over 65 years of age is increasing. Most older adults prefer to age in place, and technologies, including Internet of things (IoT), Ambient/Active Assisted Living (AAL) robots and other artificial intelligence (AI), can support independent living. P60	AI technologies	31	A user-centered design approach was used to identify older adults' perspectives regarding AAL and AI technologies and gauge interest in participating in a co-design process. A survey was used to obtain demographic characteristics and assess privacy perspectives.	Most participants identified as privacy pragmatics and fundamentalists, indicating that privacy is important to older adults. At the same time, they also reported a willingness to contribute to the design of technologies that would facilitate aging independently. There is a need to increase technology literacy of older adults along with aging literacy of technologists.

Choi J, et al. Usability testing of tablet-based cognitive behavioral intervention application to improve a simple walking activity for older adults with arthritis fatigue. Geriatr Nur (Lond). 2021;42(2):473-478. doi:10.1016/j.gerinurse.2021.02.014	quantitative	USA	The purpose of this study was to test usability of a tablet-based cognitive behavioral intervention (“Tab-CBI”) application. Tab-CBI was designed to improve a simple walking activity for older adults with arthritis fatigue. P473	tablet-based intervention/Video conferencing/ Mobile	phase I with 5 older adults to identify any initial design issues and phase II with 10 older adults to examine if Tab-CBI was seamlessly integrated into their daily living.	PROMIS Fatigue Short Form v1.0 Fatigue-8a (PROMIS F-SF)	Participants favored video-based learning and videoconferencing in the Tab-CBI program, with feedback incorporated into its final version. A pilot study is underway to test its effects on walking, fatigue, self-confidence, and quality of life in older adults with arthritis fatigue.<br>The study highlights the importance of a user-centered approach in developing health technologies to ensure usability and acceptance among older adults.
Abbaspur-Behbahani S, et al. Application of mobile health to support the elderly during the COVID-19 outbreak: a systematic review. Health Policy Technol. 2022;11(1):100595. doi:10.1016/j.hlpt.2022.100595	Systematic Review						
Li G, et al. Effects of virtual reality-based interventions on the physical and mental health of older residents in long-term care facilities: a systematic review. Int J Nurs Stud. 2022;136:104378. doi:10.1016/j.ijnurstu.2022.104378	Systematic Review						
Zhu X, et al. How tablets/applications enhance social connections and social support in people with dementia: a qualitative systematic review. Int J Ment Health Nurs. 2023;32(3):727-743. doi:10.1111/inm.13112	Systematic Review						

Bertolazzi A, et al. Barriers and facilitators to health technology adoption by older adults with chronic diseases: an integrative systematic review. BMC Public Health. 2024;24:506. doi:10.1186/s12889-024-15477-1	Systematic Review						
Dasgupta D, et al. A survey of tablet applications for promoting successful aging in older adults. IEEE Access. 2016;4:9005-9017. doi:10.1109/ACCESS.2016.2632818	Scoping Review						
Palacios-Ceña D, et al. Long-term care facilities and nursing homes during the first wave of the COVID-19 pandemic: a scoping review of the perspectives of professionals, families and residents. Int J Environ Res Public Health. 2021;18(19):10099. doi:10.3390/ijerph181910099	Scoping Review						
Hung L, et al. Using touchscreen tablets to support social connections and reduce responsive behaviours among people with dementia in care settings: a scoping review. Dementia (London). 2021;20(3):1124-1143. doi:10.1177/1471301220922745	Scoping Review						

Koo BM, et al. Mobile technology for cognitive assessment of older adults: a scoping review. Innov Aging. 2019;3(1):igy038. doi:10.1093/geroni/igy038	Scoping Review						
